# Gamifying Sexual Education for Adolescents in a Low-Tech Setting: Quasi-Experimental Design Study

**DOI:** 10.2196/19614

**Published:** 2021-10-12

**Authors:** Hussein Haruna, Kingsley Okoye, Zamzami Zainuddin, Xiao Hu, Samuel Chu, Samira Hosseini

**Affiliations:** 1 Writing Lab, Institute for the Future of Education Tecnologico de Monterrey Monterrey Mexico; 2 Faculty of Education The University of Hong Kong Hong Kong Hong Kong; 3 School of Engineering and Sciences Tecnologico de Monterrey Monterrey, Nuevo Leon Mexico

**Keywords:** gamified instruction, serious gaming, gamification, educational innovation, teenage students, digital generation, e-learning, low-tech setting

## Abstract

**Background:**

Sexual education has become increasingly important as unhealthy sexual practices and subsequent health risks become more prevalent during adolescence. Traditional sex education teaching methodologies are limiting for digital natives exposed to various digital technologies. Harnessing the power of technology applications attractive to the younger generation may be a useful approach for teaching sex education.

**Objective:**

The aim of this study was to improve sexual health knowledge and understanding of the problems associated with unhealthy sexual practices and address sexual and reproductive health challenges experienced in a low-tech setting.

**Methods:**

A participatory design approach was used to develop the digital gamified methodology. A sample of 120 secondary school students aged 11-15 were randomly assigned to either experimental or control group for each of the 3 teaching approaches: (1) gamified instruction (actual serious games [SG] in teaching); (2) gamification (GM; making nongames, such as game-like learning); and (3) traditional teaching (TT) methods.

**Results:**

The SG and GM approaches were more effective than TT methods in teaching sexual health education. Specifically, the average scores across groups demonstrated an increase of mean scores from the pre- to posttest (25.10 [SD 5.50] versus 75.86 [SD 13.16]; t_119_=41.252; *P*<.001 [2 tailed]). Analysis of variance indicated no significant differences across groups for pretest scores (*F*_2,117_=1.048, *P*=.35). Significant differences across groups were evident in the posttest scores. Students in the SG and GM groups had higher average scores than the TT group (*F*_2,117_=83.98; *P*<.001). Students reported increased learning motivation, attitude, know-how, and participation in learning (*P*<.001) when using SG and GM approaches.

**Conclusions:**

Digital health technologies (particularly teaching and learning through gamified instruction and other novel approaches) may improve sexual health education. These findings may also be applied by practitioners in health care settings and by researchers wishing to further the development of sex education.

## Introduction

### Background

Unhealthy sexual activity and its related diseases have increased globally. Nowhere is the effect of sexually transmitted diseases more apparent than in the countries of sub-Saharan Africa (SSA). In SSA countries, many adolescents are exposed early to sexual intercourse and sexual and emotional abuse [1]. Consequently, adolescents are vulnerable to unsafe sexual intercourse practices, sexual encounters with many partners, forced sexual contact, exploitive sexual activity and relationships, and influence from sexually active friends [2-5]. Exposure to these types of sexual practice has led to an increase in sexually transmitted infections (STIs), such as HIV/AIDS and Chlamydia [6-8], and other consequences. A plethora of research studies have supported the increase of sexual health literacy as a way to reduce the spate of unhealthy sexual practices and curb the current increase in sexually transmitted diseases [3,9-11]. A variety of sexual health education programs for adolescents have been implemented globally. However, the efficacy of pedagogy plays a crucial role in fostering sexual health knowledge acquisition. An effective pedagogy supports a host of academic achievement paradigms [7,8,12-15]. Although effective pedagogy has been given less emphasis in sexual health education than in core curriculum subjects [16], initiatives are being undertaken for more effective sexual health education in the digital era.

Digital health games designed to target sexual health practices have increasingly demonstrated their capabilities, appeal, and influence on educating digital native adolescents [17]. Gamified learning (serious games [SG] and gamification [GM]) platforms provide unique methods for delivering educational objectives, increasing knowledge, and reducing sex-related problems faced by adolescents [18-20]. The capacity of outreach for digital games is higher than that for traditional teaching (TT) methods [21]. Approximately 97% of adolescents normally engage in digital games, whereas 50% spend more than 1 hour per day on one kind of gaming equipment or platform or another. The Speak Up Project for Digital Learning revealed a higher preference for digital gamified learning platforms for instruction over traditional ways of learning [22]. When considering the sensitive nature of sexual health knowledge dissemination, digital games are attractive because they offer a discreet, interactive, and confidential environment for learning. This makes a difference for conservative societies [3,23].

Digital games facilitate role playing and offer challenging approaches to learning improving attitude and decision-making skills applicable to real-life scenarios. Because digital platforms offer an engaging approach for learners, they promote knowledge acquisition [8]. The novelty of this study is highlighted by the exploration into sexual health education in SSA countries, which have limited technology use. Only one study has investigated attitude changes and sexual health knowledge acquisition in a country with a similar low-tech environment [24]. Appeals for data supporting the use of technology to disseminate sexual health knowledge in low-tech settings have been documented [25]. This study employs a participatory research approach. It does this to design 2 digital health interventions (SG and GM) that assess 4 aspects (motivation, attitude, knowledge, and engagement [MAKE]) among adolescents in SSA countries.

### Objective of This Study

The aims of this study are to (1) add to the limited existing knowledge of game-based technologies and (2) address the interest in using this novel kind of technology as the teaching approach in a low-tech setting in Africa. First, we hypothesized that the application of game elements and mechanics in learning would enhance the sexual health literacy of teenage students. Second, we hypothesized that the teenage students would develop an attitude toward gamified instruction (SG and GM) that was more favorable and receptive than that toward the traditional learning approaches. Henceforth, this study looks at how gamified instruction can improve the sexual health education of adolescents, address their sexual health challenges, and help them overcome those challenges, all of this in developing countries, which tend to be far less invested in digital technologies than developed countries [26].

## Methods

### Study Design

**Figure 1:**
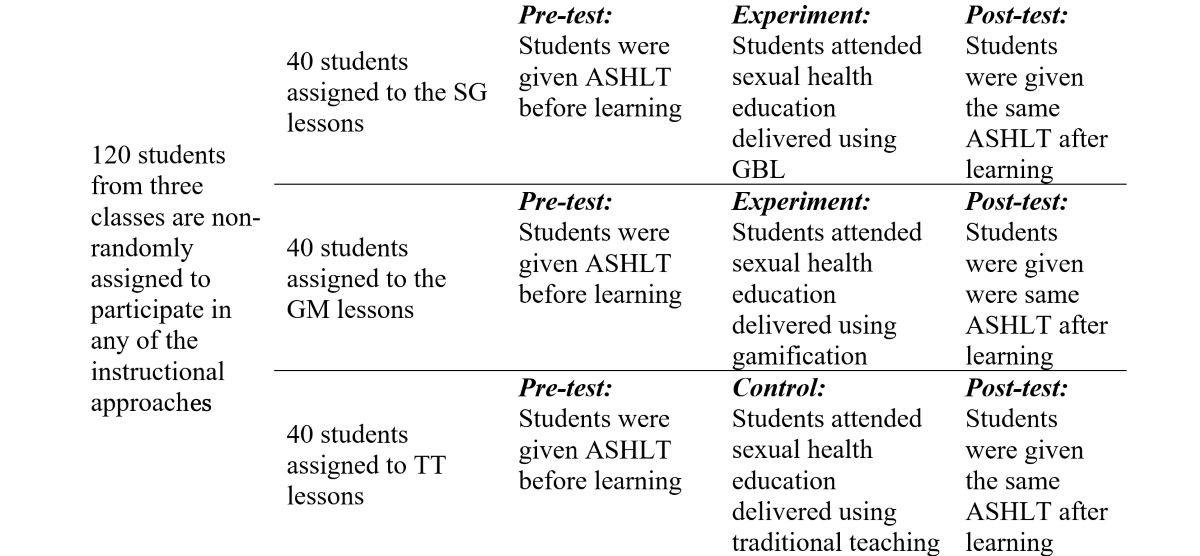
Quasi-experimental research design employed in this study. Students in their existing 3 classes were nonrandomly selected to participate in one of the 3 instructional approaches (SG, GM, and TT). ASHLT: Adolescent Sexual Health Literacy Test; GM: gamification; SG: serious game; TT: traditional teaching.

This study employed a quasi-experimental research design. The design guided the sexual health literacy interventions for students clustered in 3 classes. Sexual health education was made mandatory for all students to sanction the randomization technique [26,27]. The study was also in line with previous empirical studies and other publications that guide quasi-experimental research design [28-30]. It evaluated learning outcomes using pre- and posttest evaluations across the 3 teaching approaches (SG, GM, and TT). The students’ perceptions were compared to determine which instructional approach was the most effective in motivating students to learn, change attitudes, acquire knowledge, and to become engaged in the courses. Figure 1 presents the quasi-experimental research design employed in this study.

### Participants

The study involved teenage students (n=120) aged 11-15 who were enrolled in a secondary school at the time of the study. The research was carried out in a school in Dar es Salaam, Tanzania. This school was selected because it had 2 computer laboratories, each able to accommodate around 40 students. They had internet connectivity, a power supply, and a standby generator. The 3 classes had around 40 students each. Everyone in a class was in the same grade. The participants were not chosen randomly. Each participant was assigned to either an experimental group or a control group based on their intact classroom settings. The researchers had no authority to form or annihilate the existing study population setting. The research team randomly designated 2 of the classes as experimental (digital game and GM) and 1 class as control (traditional).

Each group was unaware of the other groups. The learning materials were the same for all the 3 groups. The only differences were in the instructional approaches. The participants were from different schools that had taken part in the revision and participatory design process of the interventions. Finally, there were 5 topics, each covered in one 40-minute class per week, as reported in Table 1.

**Table 1:** Sexual health education lessons covered per week and their time length (n=120). The columns and individual cells represent records per lesson, per week, and per class.

Topics	Week	Duration (minutes)	Serious game	Gamification	Traditional teaching
1. Personal hygiene and good manners	1	40	40	40	40
2. Sexual responsibility and decision making	2	40	40	40	40
3. Dealing with peer pressure during adolescence	3	40	40	40	40
4. Prevention of sexually transmitted infections, including HIV/AIDS	4	40	40	40	40
5. Dealing with harmful practices and sexual violence	5	40	40	40	40

### Study Conditions

#### Interventions

SG and GM interventions were developed following “activity theory” [31], “design-based research” [32], and participatory design approach [15,33]. All of these emphasize the involvement of stakeholders in developing instructional interventions for addressing the intended needs of the study population. As this was the third round of intervention testing, the games were refined based on outcomes from the second round. Students from this group shared their comments for making further improvement. The participatory research design approach employed led to the refinement of the 2 gamified interventions with a view to addressing the challenges faced by the adolescents [18,25]. While the revisions were carried out, the intended users of the systems/learning platforms and other stakeholders (eg, pediatricians; sexual and reproductive health specialists; sexual health teachers from participating schools; computer and information science specialists, including a game designer who was a computer engineer; and targeted secondary school student end users) were all involved in the study. These stakeholders were invited to participate in a series of design workshops during the refining of the intervention. This study also reports the research conducted during the third iteration. Further details of the SG and GM design and development have been published in another research [26]. The descriptions of each study condition are presented below.

#### Traditional Teaching Class

Students assigned to receive TT were treated as the control group. They were taught in a conventional classroom manner. Their teacher taught 1 day a week for 40 minutes for 5 weeks. Students were given hand-outs for further reading after each session. No digital technology was used.

#### Serious Gaming Teaching Class

**Figure 2:**
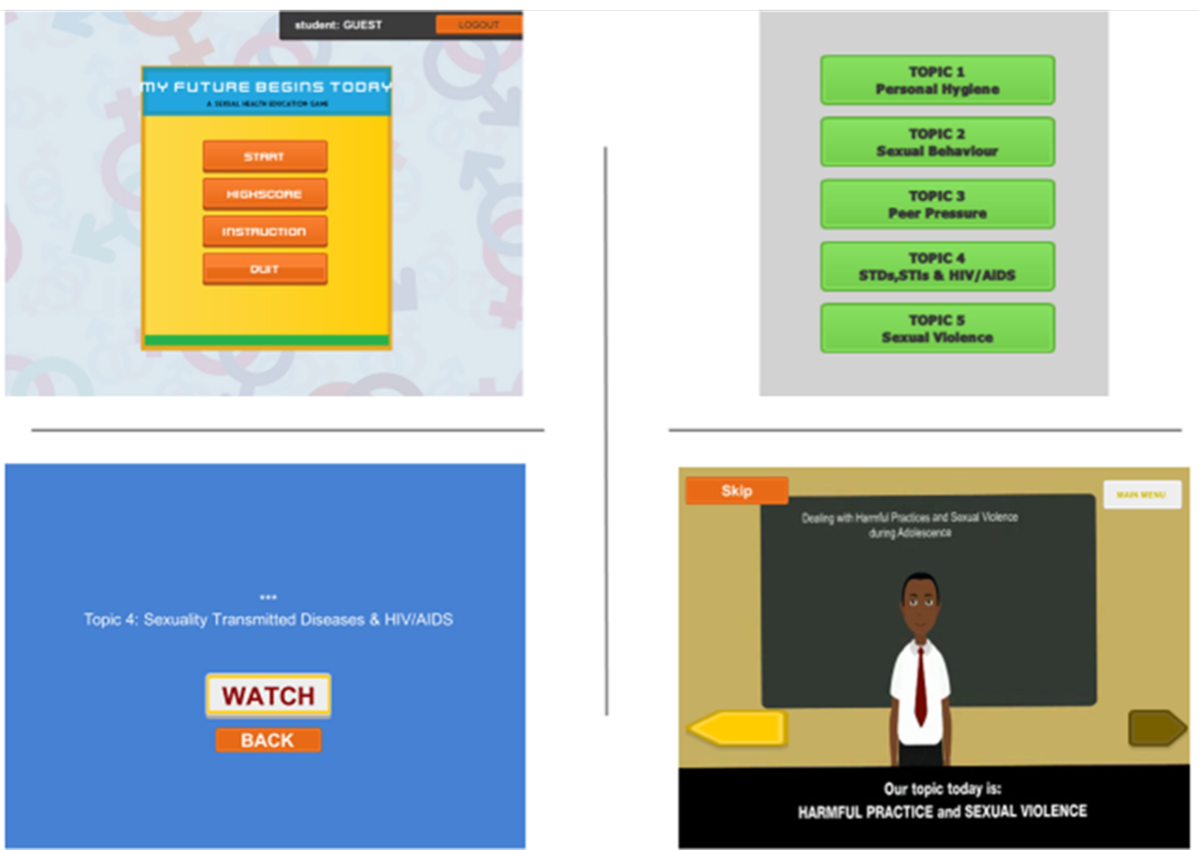
Representation of the third game platform and implementation.

**Figure 3:**
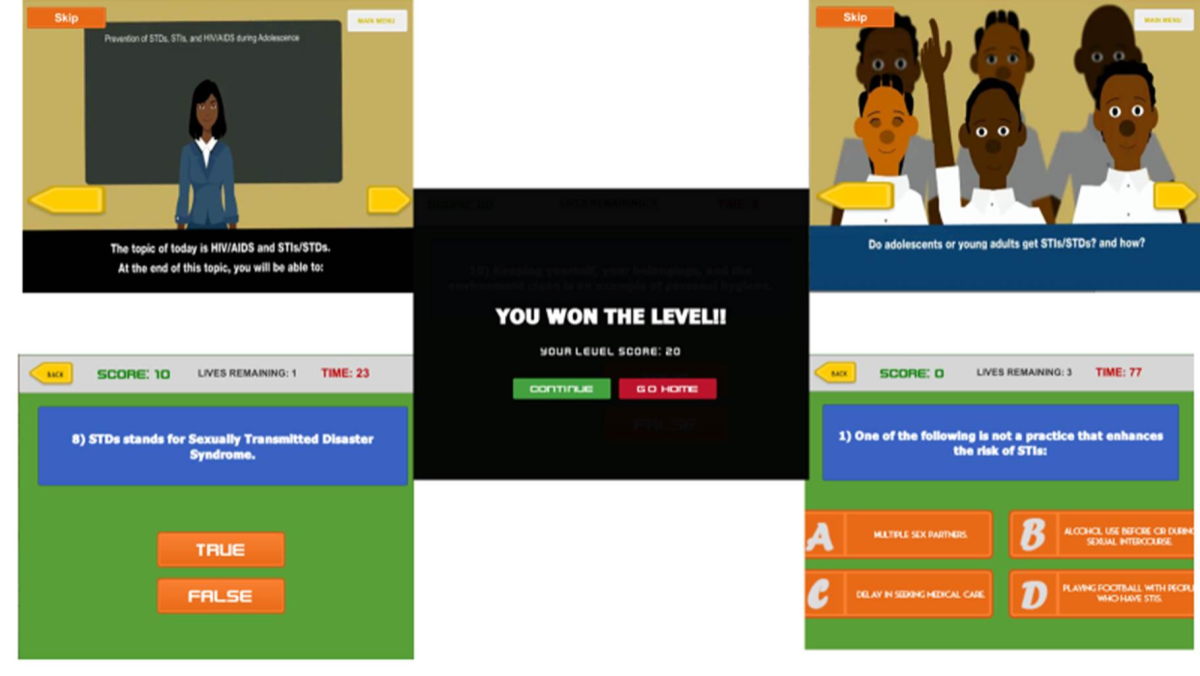
The game structures.

Students in this group received sexual health education using an SG approach (Figures 2 and 3). A week before the classes started, SG students were oriented on the game in the school computer laboratory. Students played the “My Future Begins Today” game individually under the watchful eye of a teacher and the researchers after the classes have commenced. There were 5 topics arranged in chronological order. Each topic took about 40 minutes per week. The students were also allowed to use the game during free time.

The game has an introduction which presents the general learning objective. Each topic has a game scenario in which there are avatars representing a teacher and students interacting in a classroom. There were conversations between the teacher and the students’ avatars. After watching the scenarios, the students were asked to complete the quizzes online. There were 10 quizzes on each topic, to be completed within 90 minutes. Scores were provided for correct answers, and students would lose points for each wrong answer. The students also had an opportunity to repeat the gameplay within the 40-minute margin.

#### Gamification Teaching Class

**Figure 4:**
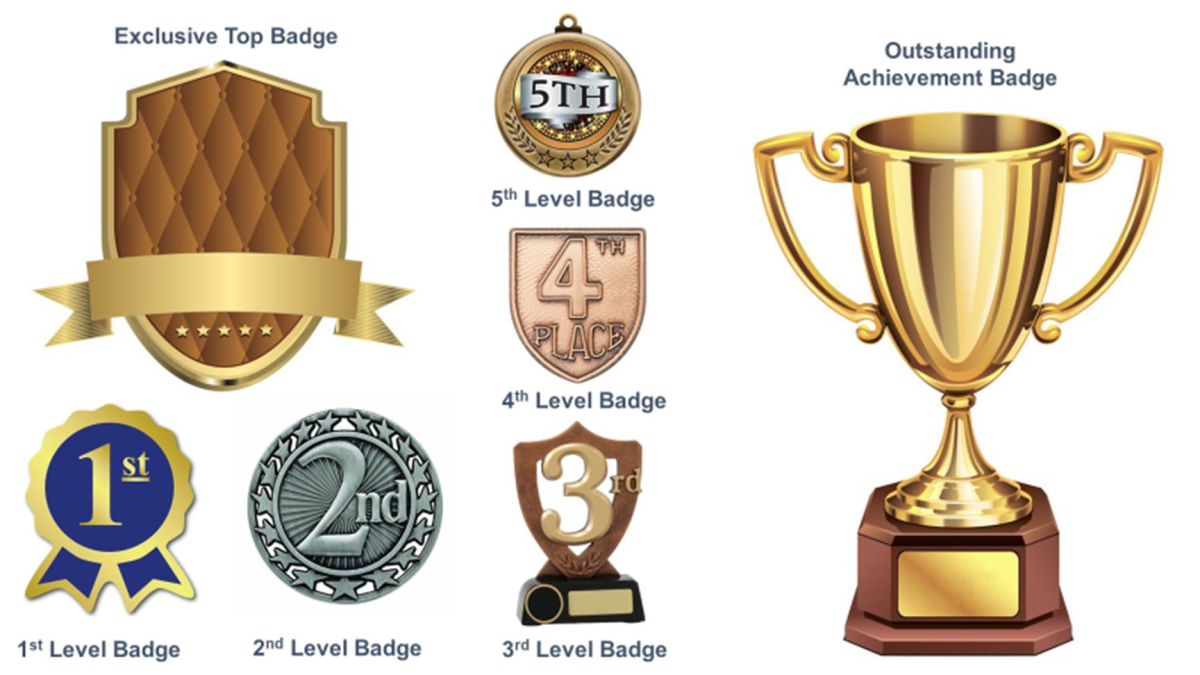
Types of badges used in the gamification group.

GM is the process of giving some of the characteristics of real games to activities that are not games. GM aims to make the learning activities more interactive. This is supposed to motivate students to learn in a way that is more effective because it is fun. GM is an emerging technique within education [3,34,35]. The concept is also a recent development in low-tech settings, especially in SSA countries. With GM, more actual learning tends to take place [36]. There are various types of learning management system platforms with built-in game mechanics [37,38]. This study used “Moodle” to organize and integrate the material we wanted to teach with game elements, such as badges, levels, leader boards, points, scores, competition, and quizzes (Figure 4). As with the SG students, the students who participated in the GM instruction received a 1-week orientation before the classes began.

There were 5 topics and for each topic 40 minutes were allocated per week for 5 consecutive weeks. Each student studied individually, but they could interact online via a discussion forum devoted to each topic. Students were asked to read and practice the lesson materials provided online. There were 10 questions under each section, some true/false and some multiple choice. There were 7 types of badges (Figure 4). One was automatically awarded upon completion of a lesson using the award rules as outlined in Table 2. Overall, the GM concept was used to make learning more fun, motivate the students to learn, support a change of attitude, and increase engagement. This study observed that learning in a competitive spirit increased the desire for continuous learning [39,40]. Students were also automatically positioned on the leaderboards and assigned levels based on the points they had gained after completing the learning activities.

**Table 2:** Criteria and rules to receive badges.

Badge name	Description
1st Level Badge	Rewarded to students who complete the first topic and are moving to the second topic
2nd Level Badge	Rewarded to students who complete the second topic and are moving to the third topic
3rd Level Badge	Rewarded to students who complete the third topic and are moving to the fourth topic
4th Level Badge	Rewarded to students who complete the fourth topic and are moving to the fifth topic
5th Level Badge	Rewarded to students who complete the fifth topic
Exclusive Top Badge	Rewarded to a student with the highest score for a particular topic
Outstanding Achievement Badge	Rewarded to a student with the highest total of points from all topics

### Procedure and Data Collection Methods

#### Sexual Health Literacy Tests

During the design of the interventions the selected research team and participating parties, especially the teachers and sexual/reproductive health specialists, were involved in developing a set of questions covering the 5 topics taught. This test was titled Adolescent Sexual Health Literacy Test (ASHLT). There were 50 questions, 10 per topic in the following format: Section A (multiple choice), Section B (true/false), and Section C (short answer). The ASHLT took up to 45 minutes to complete. Before initiating the actual learning, students were asked to do a pretest (using the ASHLT) to assess their sexual health knowledge (baseline). Within a week following the training, the same ASHLT questions were given to measure their level of understanding.

#### Students’ Perceptions of Teaching Approaches

This study used the MAKE framework [41], according to which a teaching method is regarded as effective if it shows the ability to *m*otivate students, improve their *a*ttitude, increase their *k*nowledge acquisition, and increase their *e*ngagement in learning. Several scholars have employed the method for evaluating the efficacy of the 4 components of MAKE independently [8,15,24,42-44]. This study employed a MAKE evaluation framework to evaluate and compare the efficacy of the 3 instructions by taking into account the 4 different perspectives (motivation, attitude, knowledge, and engagement) at once. The resultant MAKE instrument we used has 46 items, with the motivation construct containing 16 items and the other 3 constructs (attitude, knowledge, and engagement) having 10 each. We measured the students’ viewpoints through a self-rating method that had a 5-point Likert scale (5=strongly agree to 1=strongly disagree). The ratings took 10 minutes to complete and were all conducted within a week.

#### Focus Group Interviews

We conducted focus group interviews (FGIs) to yield more comments on the teaching methods [45]. The FGIs were conducted to corroborate and complement the quantitative data. A total of 21 students were requested to participate in the FGI, 7 students for each of the 3 learning instructions. These are realistic numbers for an FGI [46]. There were 3 focus group discussions, one for each of the instruction categories. A semistructured interview guide/protocol was adopted from the MAKE evaluation framework. Students were asked to share their views on the effectiveness and other aspects of their learning approach, and an audio record of the FGI data was made. Verbatim transcriptions were made using pseudonyms for data analysis. The participants were given equal time (1 hour) to provide their comments.

### Quantitative Data Analysis

#### Overview

The collected data were imported from the Excel (Microsoft) file format to the IBM SPSS software tool for statistics (version 24) to perform the quantitative analytic tests. This was for data generated using ASHLT and the MAKE evaluation framework. A paired *t* test was conducted to compare the pre- and posttest average scores. This was done to determine whether there were changes in the learning scores after a series of sexual health literacy sessions. Besides, a one-way analysis of variance (ANOVA) was performed to analyze the numerical data collected from the pre- and posttest scores. This test compared the variations across the 3 learning approaches. In other words, we performed the pretest comparison across the 3 instructions to establish possible significant differences before the training. This would especially rule out any possible bias in the sexual health knowledge of students collected at baseline. The descriptive statistics was focused on determining the mean, median, and SD on the self-rating scale of the measurement using the MAKE evaluation framework pertaining to the students’ perceptions of the 3 instruction approaches. The self-ratings of the effectiveness of the 3 teaching methods using the MAKE evaluation framework for each component were tested using the one-sample Kolmogorov–Smirnov and Shapiro–Wilk tests for normality. Table 3 presents the results of the normality tests.

Although the results indicated that the data samples violated the assumption of normality (*P*<.05), as the scores are non-normally distributed in the Kolmogorov–Smirnov and Shapiro–Wilk tests (Table 3), a nonparametric Kruskal–Wallis test was consequently used to compare and contrast the responses across the 3 groups for each component. A significant value of *P*<.05 was used to determine the results of the statistical analysis.

**Table 3:** Normality test results for the MAKE instrument.

Construct	Kolmogorov–Smirnov				Shapiro–Wilk			
	Statistic	*df*	*P* value^a,b^	Statistic		*df*	*P* value^a,b^	
Motivation	.085	120	.03	.956		120	<.001	
Attitude	.213	120	<.001	.800		120	<.001	
Knowledge	.174	120	<.001	.896		120	<.001	
Engagement	.103	120	.003	.961		120	.002	

^a^All *P* values are <.05, and thus significant.

^b^Lilliefors significance correction.

#### Measurement Reliability

The validated instruments and reported questionnaires appeared to be satisfactory [41,47] following the factor analysis and reliability checks we conducted and documented for 120 samples. The sample size met the minimum of 100 or larger, or a ten-to-one ratio of observations per domain [48]. The motivation questionnaires showed a Cronbach α of .92. The attitude questionnaire showed a Cronbach α of .90, and the knowledge survey developed from the sexual health education syllabus showed a Cronbach α of .92. The engagement questionnaires developed from many sources with no existing reliability results indicated Cronbach α of .90. The results of the different scale reliability checks are presented in Table 4.

**Table 4:** Scale reliability for the MAKE evaluation instrument (N=120).

Constructs and components		Number of items	Cronbach α	Standardized α	Kaiser–Meyer–Olkin	*P* value
**Motivation**				.92	.88	<.001^a^
	Attitude	4	.92			
	Relevance	4	.93			
	Confidence	4	.90			
	Satisfaction	4	.85			
**Attitude**				.90	.88	<.001^a^
	Affective	5	.91			
	Cognitive	5	.89			
**Knowledge**				.92	.86	<.001^a^
	Importance	4	.93			
	Effectiveness	3	.93			
	Application	3	.89			
**Engagement**				.90	.87	<.001^a^
	Emotional	6	.91			
	Cognitive	4	.88			

^a^The mean difference is significant if *P* value is <.05.

### Qualitative Data Reliability and Analysis

The qualitative data collection instrument was developed using the MAKE evaluation. FGI transcriptions and records complemented the quantitative results. Membership checking, conformability, and validation were applied to the collected data to come up with critical comments on the sufficiency of the results for ensuring the reliability of the qualitative data. At the end of the data collection process, students were asked to review the transcripts to determine whether the transcripts presented incorporated their comments. Thus, based on the MAKE evaluation instrument, 4 themes were developed (ie, motivation, attitude, knowledge, and engagement) to enable an ample analysis of the collected data. Then, a codebook was created using the 4 MAKE constructs. The students’ transcripts were merged with the quantitative data (based on the 4 MAKE themes).

## Results

### Baseline Characteristics of Participants

In all, 120 teenage students participated in testing the interventions. Table 5 presents their demographic features and socioeconomic status, including their access to and use of digital technologies.

**Table 5:** Descriptive characteristics of participants (N=120).

Characteristics		Value
**Sex, n (%)**		
	Male	69 (57.5)
	Female	51 (42.5)
**Age, mean (SD)**		
	Male	13.65 (0.99)
	Female	13.65 (1.01)
**Living group, n (%)**		
	With both parents	89 (74.2)
	With father only	7 (5.8)
	With mother only	16 (13.3)
	With guardian only	8 (6.7)
**Economic group, n (%)**		
	High class	14 (11.7)
	Middle high class	47 (39.2)
	Middle low class	57 (47.5)
	Poor	2 (1.7)
**Access to a computer at school or home, n (%)**		
	Yes	118 (98.3)
	No	2 (1.7)
**Access to smart devices at school or home, n (%)**		
	Yes	119 (99.2)
	No	1 (0.8)
**Play of computer or mobile phone games, n (%)**		
	Yes	117 (97.5)
	No	3 (2.5)

### ASHLT Test Results

#### Main Findings

**Figure 5:**
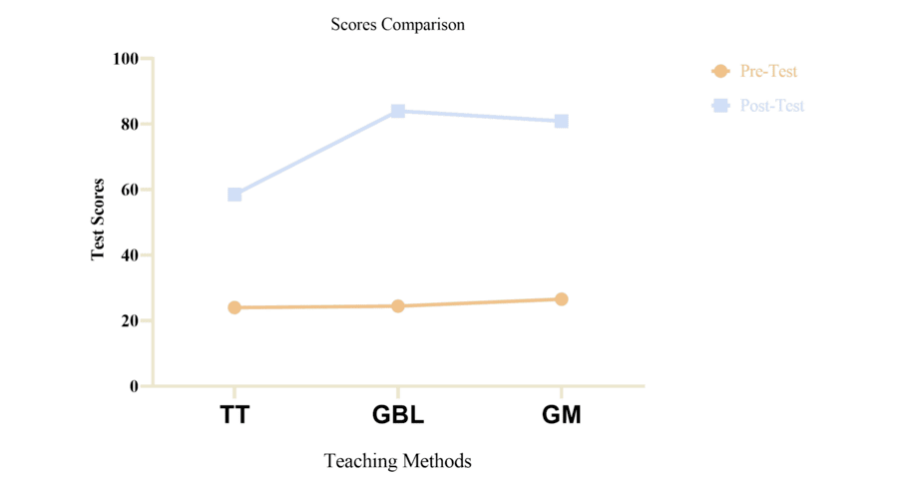
Average comparison of three teaching groups.

The study carried out a paired sample *t* test to assess the mean differences or effect of the 3 teaching methods based on the students’ average scores in the ASHLT. Statistically, there emerged a significant improvement in the knowledge acquisition, as the data demonstrated an increase in mean scores in the ASHLT from pretest mean of 25.10 (SD = 5.50) to posttest mean of 75.86 (SD 13.16; t_119_=41.252, *P*<.001; 2-tailed). A one-way ANOVA was then used to compare and contrast pre- and posttest across the 3 instructions. The average of pretest scores indicate that participants were equally distributed in all the 3 teaching methods: *F*_2,117_=1.048, *P*=.35. The average posttest scores stemming from the experimental instructions (SG and GM) also indicated an increase—as opposed to their counterparts in the control group (TT): *F*_2,117_=83.98, *P*<.001. Figure 5 presents a comparison of the effectiveness of the 3 teaching groups.

Likewise, we conducted post hoc tests (which served as follow-up analysis) to establish the differences in the 3 pairs of teaching groups: 2 experimental and 1 control. Significant divergences emerged for both the traditional and game-based groups (*P*<.001) and between the TT and GM groups (*P*<.001). However, there was no significant difference between SG and GM groups (*P*=.19). These results suggest that students assigned to the experimental groups achieved a higher score after the lessons than the students in the control group.

#### Comparison of Average Scores Before the Series of Lessons

Table 6 presents the results for all the 5 topics. For example, the descriptive data we provide below demonstrate the average scores for the “personal hygiene and good manner during adolescence” topic as follows:

- TT group mean score of 5.45 (SD 1.73)
- SG group mean score of 5.36 (SD 2.15)
- GM mean score of 5.68 (SD 2.22)

As gathered in Table 6, a one-way ANOVA was conducted to compare each sexual health education topic taught across the 3 instructions (ie, TT, SG, and GM). The results indicated a nonstatistically significant difference in any of the 5 topics detected with *P*>.05. The results suggest that all the 3 teaching methods can be considered similar regarding sexual health knowledge in all 5 topics before the training.

**Table 6:** Comparison of average score and one-way ANOVA results before the series of lessons.

Topic	Pretest, mean (SD)				One-way ANOVA
	TT	SG	GM		
1	5.45 (1.73)	5.36 (2.15)	5.68 (2.22)	*F*_2,117_=0.26, *P*=.76	
2	4.92 (1.59)	4.92 (1.45)	5.31 (1.44)	*F*_2,117_=0.89, *P*=.41	
3	4.85 (1.18)	4.87 (1.65)	5.40 (1.60)	*F*_2,117_=1.72, *P*=.18	
4	4.66 (1.16)	4.67 (1.57)	4.71 (1.15)	*F*_2,117_=0.01, *P*=.98	
5	4.76 (1.52)	4.68 (1.53)	5.01 (1.36)	*F*_2,117_=0.53, *P*=.58	

#### Comparison of Average Scores After the Series of Lessons

The students in each group also completed the same (ASHLT) quizzes as a posttest. The one-way ANOVAs were performed to evaluate the students’ average test scores across the 3 teaching methods. The descriptive data from the one-way ANOVAs and post hoc comparison tests are illustrated in Multimedia Appendix 1. The results indicated that the SG and GM groups had higher averages than the TT group.

The one-way ANOVAs revealed that the students received effective sexual health knowledge and that the acquisition rate increased for each topic in the 3 learning groups. Students from the SG and GM instruction groups showed a significant knowledge acquisition in topic 1, compared with students in the TT group (*F*_2,117_=19.04, *P*<.001). Constant effects remained for the other 4 topics. The Tukey HSD post hoc multiple comparison tests indicated that the average scores for all the 5 topics significantly varied between the control and experimental groups (*P*<.001). The experimental groups did not differ significantly as *P* values were over .05 (refer to Multimedia Appendix 1 for details).

### Students’ Perceptions Toward Instruction Approaches

The study evaluated the students’ perceptions of the 3 instructional approaches using a self-rating scale and FGI. The details of both the quantitative and qualitative results are presented below.

#### Quantitative Component

The averages for the responses to each aspect of the MAKE evaluation framework were compared for the 3 groups. A Kruskal–Wallis test was performed to determine the existence or nonexistence of statistically significant difference among the 3 groups after rating the average scores from each MAKE evaluation framework. Statistically significant dissimilarities between the 3 groups’ averages were demonstrated (Multimedia Appendix 2). For instance, the Kruskal–Wallis test showed that there was a significant difference in motivation between the 3 instructions: SG mean of 4.51 (SD 0.25), GM mean of 4.40 (SD 0.38), and TT mean of 4.12 (SD 0.59); *P*<.001. Post hoc tests were also conducted to make pairwise comparisons. In the post hoc tests, we found that TT differed significantly from both GM (*P*=.04) and SG (*P*<.001). By contrast, GM and SG were not significantly different from each other (*P*=.79). Moreover, this effect remained consistent with the other aspects of the MAKE evaluation.

#### Qualitative Component

Comments were received from both the experimental and the control groups on the 3 instruction methods. Like the results from the quantitative data, students from the experimental groups commented favorably on the SG and GM instructions, whereas those in the control group commented unfavorably on TT. For example, for *motivation*, the students reported:

the games were fun

SG-3

...that learning was easy

SG-5

...the learning offered a self-regulatory method that improved my confidence

GM-1

...learning was [done] in a competitive [way], which helped me gain problem solving-skills

GM-7

Students also pointed out that

...the learning inspired me; hence I focused on learning

SG-2

...I was extremely interested in the learning approach

GM-4

...the availability of badges encouraged [me to learn] the subject

GM-2

By contrast, students from the TT group were largely negative about their learning experience:

...there were no visuals

TT-5

...[there was] no clarification on many issues

TT-2

...[there were] limited, or no activities for concentration

TT-6

...little or none of the critical thinking strategies were provided, including role play, demos, quizzes, team-work activities, [or] collaboration.

TT-3

Regarding the *attitude change*, the FDIs revealed the opinion of the experimental group students about SG and GM:

...particularly useful in changing attitudes

GM-3

...a non-embarrassing learning environment

SG-1

...suitable and worthwhile for the delivery of sexual health education

SG-7

I was excited about the activities, competitions, leader-boards, badges, avatars, and scenarios

GM-4

By contrast, the control group students commented:

I was bored listening to lectures

TT-1

...[it was] hard to understand how the sexual health subject is important for changing my attitude

TT-7

...[it is] unfriendly learning

TT-6

...[it was an] uncomfortable learning environment due to the sensitivity of the topic taught

TT-5

...it hides potential information for changing my attitude

TT-4

...questions were not encouraged or not well clarified; hence I ended up with no clues that could change the myths [that produce] negative sexual health attitudes

TT-2

Experimental group students reported having had positive interactions with the SG and GM interventions and a substantial improvement in their sexual health *knowledge*:

I acquired the required knowledge for practicing healthy sexual behaviours through this learning approach

GM-6

I acquired potential sexual health knowledge that will help my making informed decisions

SG-6

I will apply the skills and understanding that are essential and applicable for curbing unhealthy sexual behaviours.

SG-4

from today onwards I will not participate in risky sexual activities as I am [now] knowledgeable and will apply the knowledge acquired to make informed decisions for better sexual health outcomes and future goals.

SG-4

By contrast, control group students commented on their teaching method as follows:

traditional teaching was less informative

TT-3

technical language was used that made it difficult to understand

TT-7

[there was a] lack of vivid examples

TT-1

the learning strategy narrowed the thinking capacity required for applying the knowledge and skills acquired

TT-4

Finally, students in the experimental learning groups (SG and GM) reported that the SG and GM components were effective in *engaging* them:

the learning activities made our minds active

GM-6

I was connected to the learning process

SG-4

I focused on the learning activities

SG-4

the learning made me concentrate on learning all the time

GM-2

Students in SG-2 and GM-7 reported that

...the learning provided opportunities for hands-on activities that made it easy to learn and remember.

By contrast, the TT students reported that

our learning was indirect

TT-3, TT-5, TT-2

the learning was passive, as no hands-on activities were provided

TT-1, TT4

there was little or poor interaction

TT-7

I lost focus during learning

TT-6

## Discussion

### Findings and Interpretation

The study showed that the game elements embedded in SG and GM instruction catalyzed motivation and engagement during learning and that this contributed to attitude change, knowledge acquisition, and ultimately better learning performance.

The study results demonstrated that sexual health education taught using SG and GM approaches works better than TT methods. The SG and GM approaches resulted in higher test scores for knowledge acquisition than the TT control group. This finding conforms with previous research which found gamified learning systems to have a significant impact on sexual health education [25]. In our study, most students acknowledged several factors in their improved learning: The first factor is motivation (to learn), which consists of elements of attention, relevance, confidence, and satisfaction [49]. Indeed, motivation is a significant component for accomplishing or failing a task [50]. Students reported that their interest was caught and improved with the game elements (scenarios, quizzes, competition, challenges, scores) provided during the learning process. The game elements motivated the students to learn [45]. Furthermore, the game elements made the learning more interactive and fun, which increased the students’ motivation to learn [50].

This study found that gamified learning contents were experienced as “relevant.” For instance, the students realized that there was a common connection between what they were learning and real life. These results were consistent with an earlier study [19], which had indicated that gamifying sexual health learning approaches was promising for adolescents because the role plays and scenarios reflect the actual lifestyle of the current generation. The students felt confident while going through the self-regulated learning material provided through the gamified learning (which stimulated and sustained their learning). Perhaps this means that they would succeed in learning the subject matter to a great extent. As this paper demonstrates, such confidence enabled them to succeed and derive self-esteem from the knowledge they acquired and apply it in real life [49]. Although Keller [49] did not examine the effectiveness of the mediating role of increased knowledge in sexual health literacy, this work showed that sexual health education interventions through gamified learning are effective for the development of self-efficacy. As a result, they encourage healthy sexual practices including the digitally savvy adolescents [24]. Students reported satisfaction with their learning experience. The quality of the gamifying content gave them an experience of fun learning (thus, accomplishing learning goals). The awards and scoring mechanics also inspired them to learn with persistence and intensity [51].

The second factor accounting for the effectiveness of the gamified learning was that it changed the attitude of the students. As a similar study [7] reported, gamified learning induces positive changes in the sexual health attitude of adolescents. Essentially, the My Future Begins Today gamified learning incorporated in its design most of the known relevant features that have proved effective in transforming adolescents’ negative sexual health attitudes to positive ones and, as a consequence, curbing risky sexual behavior [10]. It considered specific settings and co-opted various stakeholders, including the targeted users (high-school students and their teachers) in the design.

During the gamified design and development, students provided input on the type of avatars and scenarios they found appealing. Their opinions were based on their different cultural settings, their level of digital literacy, their use of state-of-the-art technology, among others. This study was grounded on the social–cultural theory known as activity theory that encourages participation of different stakeholders in the development of instructional interventions [52-54]. Members of the community participated in the design process by contributing to the design of the knowledge-acquisition components useful in addressing the problems related to acquisition of sexual health knowledge among adolescents in the studied region. The resultant gamified learning elements were found to be relevant in changing the students’ attitude toward problems such as negative peer pressure, teen pregnancy, STIs (including HIV/AIDS), and sexual violence. Although this research found no participatory design being applied within the TT environment, the gamified learning instructions invited the targeted users to participate in the design. This allowed us to apprehend the participants’ relevant ideas and needs and in turn effectively deliver the sexual health information required to induce a change in attitudes of the participants [15].

The third factor accounting for the efficacy of gamified learning was that it improved the knowledge acquisition among students in the experimental groups. The students indicated that gamified learning helped them to acquire sexual health information and skills that could purportedly help them engage in healthy sexual practices. Notably, the students reported that the sexual health knowledge delivered through gamified instructions were highly effective for their current and future lives and that they now felt knowledgeable and able to resist detrimental sexual health risks or factors. According to Chu et al [15], gamified learning is effective because knowledge is acquired in a safe, nonrisk (simulated) environment. Gamified learning offers students the opportunity to experiment in their learning, play, apply decision-making skills, and test scenarios without negative consequences. Evidence from follow-up studies shows that the effect of sexual health knowledge acquired through gamified learning is compelling and persistent, but no effectiveness has been demonstrated in terms of delay in sexual initiation [7], although the determination of outcomes was based on self-reporting. An iterative design study with the objective to describe a methodology for developing an SG intervention for improving sexual health education among youth in Boston [8] demonstrated that nonidentified study participants (ie, students and street youths) in underserved communities would have acquired more knowledge on chlamydia because they enjoyed the gameplay and actively participated in acquiring the information. This may explain why the experimental groups in our study showed better results than their counterparts in the control group.

The fourth and final factor that boosted students’ learning was engagement. The evaluation of the effectiveness of each of the instructional approaches was based on the students’ engagement. Studies have documented the effectiveness of gamified learning in engaging students during learning [8,42,43], with some studies related to the ability of gamified instructions to entertain and reduce stress when learning [43,50]. The My Future Begins Today (GM and SG) platform was designed to trigger students’ engagement by having learning tasks performed in a problem-solving way and by having students participate in skills-based challenges that required critical thinking. The gamified learning platforms are useful for the students, especially when it comes to (1) the skills that are needed to thrive or (2) use the latest technologies of this century. The presence of game elements (badges, score, leaderboards, levels, immediate feedback, time pressure, and repetition) may explain why gamified learning increased the engagement of students in the experimental group, which, in turn, bolstered their learning. Besides, game elements were positively commented on by the students who saw increased engagement with the PR:EPARe game [42]. Although Jiang et al [8] did not find a significant correlation between the participants’ game engagement and learning, this study shows that the 2 concepts are useful for learning purposes.

### Limitations

This study used a participatory design approach. Such an approach is vital in designing instructions that address the needs of the users [15,43] in their social–cultural context. It was informed by design-based research (from a technology perspective) [32] and grounded in sociocultural learning theory [53,54]. As good as the foundations are, we must acknowledge some limitations in our efforts to put them into practice. This study evaluated knowledge acquired by the students and the effectiveness of that knowledge in changing their attitudes toward sexual activities. However, it is would be difficult to determine how much of and for how long the change took place after the study. Hence, would need to know how many students dropped out of school due to pregnancy, were infected with STIs, encountered sexual violence, or were peer pressured into harmful sexual practices. A follow-up study could be conducted when the students are about to finish their ordinary-level studies.

### Conclusions

Educational gamified learning (GM and SG) has the potential to significantly increase the sexual health literacy of adolescents. The digital health gamified interventions designed in this study provide a user-friendly learning environment. The designs were influenced by a theory-driven assessment of learning. This assessment is supported by testing the learning of the users. This study treats serious digital health gamified instructions as a brain activator: it keeps students active during the learning process. The students’ participation in the learning process is catalyzed by the *motivation* and *engagement* that are enabled by the game elements and mechanics. The My Future Begins Today (the digital health gamified learning interventions using the SG and GM) design increases knowledge acquisition and attitude change. Students reported the learning to be more interactive through participating in the gamified learning activities.

SG and GM methods were found to be effective and efficient in increasing motivation, improving attitudes, increasing knowledge acquisition, and encouraging engagement in the learning process. Future empirical studies may verify the efficacy of the My Future Begins Today learning platforms in improving sexual health literacy acquisition in other countries, especially in SSA, where the TT method is widely practiced and tends to limit the learning with gamified digital technologies and process [19,25]. This paper also addresses more than ever the call from a previous study [25] to evaluate the effect of gamifying sexual health education when different key stakeholders are involved in the design process in low-tech settings. This is due to the fact that in developing countries information and communication technologies and digital literacy are limited.

## Appendix

Multimedia Appendix 1 Comparison of average scores, one-way ANOVA, and post hoc comparison tests after the lessons.

Multimedia Appendix 2 Evaluation of the efficacy of the instructional approachesa.

## Abbreviations

ASHLT: Adolescent Sexual Health Literacy Test
AVOVA: analysis of variance
FGI: focus group interview
SG: serious game
GM: gamification
MAKE: motivation, attitude, knowledge, and engagement
SSA: sub-Saharan Africa
STI: sexually transmitted infections
TT: traditional teaching
