# Supplementary material for: Gamifying Sexual Education for Adolescents in a Low-Tech Setting: Quasi-Experimental Design Study
Source: JMIR Serious Games. 2021 Oct 12;9(4):e19614. doi: 10.2196/19614 (PMC8548970; doi:10.2196/19614)
Supplement: Multimedia Appendix 1 [file games_v9i4e19614_app1.docx]

| Topic and measure | | Posttest | | | One-way ANOVA | Post hoc comparison | | | |
| --- | --- | --- | --- | --- | --- | --- | --- | --- | --- |
|  |  | TT | SG | GM |  | TM1 |  | TM2 | *P* value |
|  | |  |  |  |  |  |  |  |  |
| **1** | |  |  |  | *F*_2,117_=19.04, *P*<.001^a^ |  |  |  |  |
|  | Mean | 13.46 | 16.76 | 15.95 |  | TT | < | SG | <.001^a^ |
|  | SD | 3.07 | 2.19 | 2.09 |  | TT | < | GM | <.001^a^ |
|  | Median | 14 | 17.25 | 16 |  | SG | = | GM | .31 |
| **2** | |  |  |  | *F*_2,117_=52.95, *P*<.001^a^ |  |  |  |  |
|  | Mean | 11.93 | 17.13 | 15.96 |  | TT | < | SG | <.001^a^ |
|  | SD | 2.83 | 2.01 | 2.18 |  | TT | < | GM | <.001^a^ |
|  | Median | 12 | 17.50 | 16 |  | SG | = | GM | .77 |
| **3** | |  |  |  | *F*_2,117_=59.583, *P*<.001^a^ |  |  |  |  |
|  | Mean | 12.36 | 17.43 | 16.45 |  | TT | < | SG | <.001^a^ |
|  | SD | 2.48 | 1.95 | 2.12 |  | TT | < | GM | <.001^a^ |
|  | Median | 12 | 18 | 17 |  | SG | = | GM | .12 |
| **4** | |  |  |  | *F*_2,117_=52.61, *P*<.001^a^ |  |  |  |  |
|  | Mean | 12.59 | 17.00 | 17.05 |  | TT | < | SG | <.001^a^ |
|  | SD | 2.55 | 2.23 | 1.86 |  | TT | < | GM | <.001^a^ |
|  | Median | 13 | 18 | 17 |  | SG | = | GM | .99 |
| **5** | |  |  |  | *F*_2,117_=41.64, *P*<.001^a^ |  |  |  |  |
|  | Mean | 11.43 | 16.26 | 15.84 |  | TT | < | SG | <.001^a^ |
|  | SD | 2.60 | 2.82 | 2.43 |  | TT | < | GM | <.001 |
|  | Median | 11 | 16 | 17 |  | SG | = | GM | .75 |

^a^The mean difference is significant if *P* value is <.05.
