# Supplementary material for: Gamifying Sexual Education for Adolescents in a Low-Tech Setting: Quasi-Experimental Design Study
Source: JMIR Serious Games. 2021 Oct 12;9(4):e19614. doi: 10.2196/19614 (PMC8548970; doi:10.2196/19614)
Supplement: Multimedia Appendix 2 [file games_v9i4e19614_app2.docx]

| MAKE tool | | TT | SG | GM | Kruskal–Wallis test | Pairwise comparison | | | *P* value |
| --- | --- | --- | --- | --- | --- | --- | --- | --- | --- |
|  |  |  |  |  |  | TM1 |  | TM2 |  |
|  | |  |  |  |  |  |  |  |  |
| **Motivation** | |  |  |  | *P*<.001^b^ |  |  |  |  |
|  | Mean | 4.12 | 4.51 | 4.40 |  | TT | = | GM | .04^b^ |
|  |  |  |  |  |  | TT | < | SG | <.001^b^ |
|  | SD | 0.59 | 0.25 | 0.38 |  | GM | = | SG | .79 |
| **Attitude** | |  |  |  | *P*<.001^b^ |  |  |  |  |
|  | Mean | 4.24 | 4.74 | 4.78 |  | TT | = | SG | <.004^b^ |
|  |  |  |  |  |  | TT | < | GM | <.001^b^ |
|  | SD | 0.64 | 0.20 | 0.22 |  | SG | = | GM | >.99 |
| **Knowledge** | |  |  |  | *P*<.001^b^ |  |  |  |  |
|  | Mean | 4.24 | 4.58 | 4.63 |  | TT | = | SG | .01^b^ |
|  |  |  |  |  |  | TT | < | GM | <.001^b^ |
|  | SD | 0.48 | 0.18 | 0.26 |  | SG | = | GM | .66 |
| **Engagement** | |  |  |  | *P*<.001^b^ |  |  |  |  |
|  | Mean | 4.17 | 4.50 | 4.46 |  | TT | < | GM | <.001^b^ |
|  |  |  |  |  |  | TT | < | SG | <.001^b^ |
|  | SD | 0.29 | 0.15 | 0.31 |  | GM | = | SG | .50 |

^a^A self-rating scale was used to rate each item on a 5-Point Likert scale ranging from 1 (strongly disagree) to 5 (strongly agree).

^b^*P*<.05.
